# Do Individuals with Depression and Comorbid Medical Conditions Receive Poorer Mental Health Care than those with Depression Alone?

**DOI:** 10.23889/ijpds.v1i1.390

**Published:** 2017-04-19

**Authors:** Joseph Puyat, Arminee Kazanjian

**Affiliations:** 1 University of British Columbia School of Population and Public Health

## Objectives

The prevalence of depression is 2 to 3 times higher in individuals with comorbid medical condition (CMC) than in the general population. When untreated, depression results in increased mortality, higher health care costs, greater functional disability, decreased quality of life and lower adherence to treatment regimens for the CMC. Currently, studies that examined whether depression care in those with CMC is better or worse compared to those with depression alone show inconsistent results. Furthermore, studies that compare depression care in those with specific CMCs are scarce. Improving knowledge base in this area will enable health care systems to better allocate limited resources to clinical population that need them most.

## Aims

1) To estimate disparities in depression care in those with one or more CMCs, and 2) to examine if depression care patterns in those with CMCs have been impacted by recent provincial policies.

## Method

We retrospectively examined data from physician claims, hospital separations, vital statistics, and insurance plan registries. Using this linked data, we identified dynamic cohorts of individuals with depression and CMCs in 2005 and 2012, based on the earliest date of depression diagnosis. Each cohort had exactly 12 months of lookback period for case ascertainment and 12 months of follow-up for tracking depression care patterns. The following indicators were tracked: 1) receipt of any psychological therapy, 2) receipt of any antidepressants (AD), 3) receipt of any depression treatment, 4) number of GP visits, 5) GP continuity of care index for all visits, 6) GP continuity of care for mental health visits, 6) counts of psychological therapy sessions, 7) proportion of days covered for AD, and 8) continuous medication gap for AD. Disparities in depression care were examined used generalized linear regression models.

## Results and Conclusions

Use of depression care in individuals with one or more CMC were higher across most indicators we examined except for AD initiation and GP visits for mental health reasons. In specific CMCs like cerebrovascular disease and diabetes, depression care in some areas were lower. Overall, depression care patterns seemed unchanged after the introduction of relevant provincial policies, although improvements over time appeared to have been made in certain areas.

